# Type 2 Diabetes Mellitus in Class II and III Obesity: Prevalence, Associated Factors, and Correlation between Glycemic Parameters and Body Mass Index

**DOI:** 10.3390/ijerph17113930

**Published:** 2020-06-02

**Authors:** Erika Aparecida Silveira, Lorena Pereira de Souza Rosa, Annelisa Silva e Alves de Carvalho Santos, Camila Kellen de Souza Cardoso, Matias Noll

**Affiliations:** 1 Post-Graduate Program in Health Sciences, School of Medicine, Federal University of Goiás, Goiás 74605-050, Brazil; lorenapsrosa@yahoo.com.br (L.P.d.S.R.); annelisanut@gmail.com (A.S.e.A.d.C.S.); camilacardoso_nut@hotmail.com (C.K.d.S.C.); matiasnoll@yahoo.com.br (M.N.); 2 Federal Institute of Goiás, Goiânia Oeste Campus, Goiás 74270-040, Brazil; 3 United College of Campinas, Goiás 74535-040, Brazil; 4 Nutrition Course, School of Social and Health Sciences, Pontifical Catholic University of Goiás, Goiás 74605-010, Brazil; 5 Instituto Federal Goiano, Ceres Campus, Goiás 76310-000, Brazil

**Keywords:** severe obesity, diabetes, insulin resistance, food consumption, whole grains, HOMA-IR

## Abstract

Despite the worldwide growth of class II and III obesity, the factors associated with type 2 diabetes mellitus (T2DM) in these obese individuals are not widely understood. Moreover, no study has investigated these associations in South America. Our study aimed to investigate the prevalence of T2DM and its associated factors, with an emphasis on biochemical parameters and eating habits, in class II and III obese individuals. We also aimed to analyze the correlation between glycemic parameters and body mass index (BMI). Baseline data from a randomized clinical trial (DieTBra Trial) of 150 class II and III obese individuals (BMI > 35 kg/m^2^) was used. An accelerometer, Food Frequency Questionnaire, and bioimpedance analysis were used to assess physical activity levels, eating habits, and body composition, respectively. Blood was collected after 12 h of fasting. Hierarchical multivariate Poisson regression was performed, and prevalence ratios (PRs) were calculated. Correlations between glycemic parameters (fasting blood glucose, glycosylated hemoglobin, homeostasis model assessment of insulin resistance (HOMA-IR), and insulin) and BMI were also analyzed. The prevalence of T2DM was 40.0% (95% CI, 32.1–48.3), high fasting blood glucose level was 19.33% (95% CI, 13.3–26.6), and high glycosylated hemoglobin was 32.67% (95% CI, 25.2–40.8). Age ≥ 50 years (PR = 3.17, 95% CI, 1.26–7.98) was significantly associated with T2DM; there was a positive linear trend between age and T2DM (*p* = 0.011). Multivariate analysis showed an association with educational level (PR = 1.49, 1.07–2.09, *p* = 0.018), nonconsumption of whole grains daily (PR = 1.67, 1.00–2.80, *p* = 0.049), and high HOMA-IR (PR = 1.54, 1.08–2.18, *p* = 0.016). We found a high prevalence of T2DM and no significant correlations between BMI and glycemic parameters.

## 1. Introduction

Diabetes and obesity are considered serious public health concerns worldwide, especially with the exponential growth of class II and III obesity in recent decades [1,2,3,4]. Approximately 463 million people worldwide are diabetic, of whom 90% have type 2 diabetes mellitus (T2DM) [4]. These diseases are independently known to cause serious health complications, with risks of cardiovascular diseases, higher mortality, low quality of life, and increased public health spending; however, when these disease occur concurrently, their negative effects on health, health systems, and mortality are even greater [1,3,4]. There are few studies on T2DM in obese individuals [5,6], and even fewer on those with higher obesity levels, such as class II and III [7]. The few studies on T2DM associated with higher obesity classes are related to surgical procedures [8,9,10].

In this context, it is important to understand the factors associated with T2DM, especially in class II and III obesity, as these classes have presented the greatest growth. It is also relevant to understand if body composition [5] and food consumption variables [11,12,13] play a role in the analysis of associated factors. Eating behavior is an important environmental factor to consider in obesity, as the evaluation of food groups is essential [11]. Another aspect to be analyzed is whether increased body mass index (BMI) can modify glycemic parameters in these higher obesity categories. More studies using these approaches and further information on these aspects will be important to increase knowledge about T2DM in class II and III obesity and improve prevention, control, and treatment protocols in different clinical settings [12,14].

Despite the relationship between T2DM and obesity, few studies have investigated the extent of the problem in class II and III obesity and whether food consumption, body composition, biochemical/glycemic parameters, and higher BMI increase the risk of T2DM in this population (BMI ≥ 35 kg/m^2^) [7,12], especially in patients not undergoing bariatric surgery. When reviewing the existing literature, we found that studies of this association in South American populations were lacking, despite this population presenting an increased prevalence of class II and III obesity over the last decade [1]. Therefore, the objectives of the present study were to investigate the prevalence of T2DM and its associated factors, with an emphasis on biochemical parameters and eating habits, in class II and III obese individuals. Furthermore, we aimed to analyze the correlation between glycemic parameters and BMI. The results of this study may support clinical guidelines and public health policies targeting class II and III obesity.

## 2. Material and Methods

### 2.1. Study Design and Participants

This study used baseline data from the randomized clinical trial entitled “Effect of nutritional intervention and olive oil in severe obesity (DieTBra Trial),” registered on the ClinicalTrials.gov platform (NCT02463435). Further methodological details can be found in DieTBra Trial articles with different outcomes, published elsewhere [2,15,16,17].

All class II and III obese individuals (BMI ≥ 35 kg/m^2^) from the Brazilian Unified Health System (UHS) of primary healthcare were referred to the Nutrition and Severe Obesity Clinic (ANOG/HC/UFG). At the time of study, ANOG was the only reference ambulatory to treat class II and III obesity in the metropolitan region of Goiânia, in the Midwest of Brazil. All obese class II and III individuals from UHS were invited to participate in the DieTBra Trial study and eligibility criteria were applied. The inclusion criteria were BMI ≥ 35kg/m^2^, both sexes, age between 18 and 64 years, and a residence in the city or in the metropolitan region of Goiânia, Brazil. The exclusion criteria were use of insulin and/or a medical diagnosis of type 1 diabetes, bariatric surgery, pregnancy and/or lactation, special needs, use of antiobesity drugs with a body weight reduction of >8% in the previous 3 months, nutritional or medical monitoring for weight loss or some type of nutritional treatment, use of a pacemaker, and presence of metal rods and/or screws in the body. Therefore, the individuals selected for this study constitute a representative group of individuals with class II or III obesity from the primary healthcare system of UHS.

### 2.2. Sociodemographic Data, Lifestyle, and Eating Habits

Information on educational level (years of study), economic class, marital status, color, age, and sex was collected. Lifestyle variables included smoking habits (smoker/ex-smoker and nonsmoker/never smoked) and level of physical activity (PA).

The PA level was assessed using an Actigraph accelerometer model wGT3X (Actigraph, Pensacola, FL, USA) positioned on the posterior side of the nondominant wrist. The participants were individually instructed to use the device 24 h a day for 6 consecutive days. ActiLife 6 software (Actigraph, Pensacola, FL, USA) was used for data processing. The recommendation to classify the PA level was ≥150 min/week of moderate to vigorous aerobic PA. The accelerometer provided the daily mean number of minutes spent on moderate and vigorous PA, which was multiplied by 7 days a week to obtain the mean time of moderate and vigorous PA per week lasting at least 10 min [18].

The Food Frequency Questionnaire (FFQ) was used to assess eating habits, which included details about the type of food consumed within each food group [19]. Sugary drink consumption included soft drinks, fruit nectar, and powdered drinks with no differentiation between types (diet/light or conventional).

### 2.3. Body Composition and Anthropometry

Body weight was measured in kilograms using a digital electronic scale (Welmy^®^, Santa Barbara d’Oeste, SP, Brazil), with a capacity of up to 200 kg and an accuracy of 100 g. Height was measured using a stadiometer coupled to the scale with an accuracy of 0.1 cm [20]. The BMI classifications used were recommended by the World Health Organization [21].

Obesity was assessed using bioimpedance analysis (BIA) with the Inbody S10^®^ multifrequency tetrapolar bioimpedance analyzer (Inbody, Gangnam-Gu, Seoul, Republic of Korea) [22]. All participants were instructed on the proper preparation and performance of the exam by receiving a folder containing the necessary information, such as to maintain hydration levels on the day of the exam, not to do vigorous PA the day before, and not to consume coffee, tea, cola, energy, or alcoholic drinks the day before [23]. The variables of body composition (total fat percentage, total fat mass in kilograms, and fat-free mass in kilograms) were categorized into quartiles according to distribution differences by sex.

### 2.4. Biochemical Tests and Health Condition

Blood samples were collected after a 12 h fast by a qualified professional, following high-quality standards both in the collection and analysis of the material. The lipid profile was analyzed using the following variables: hypercholesterolemia (total cholesterol ≥ 200 mg/dL), hypertriglyceridemia (triglycerides ≥ 150 mg/dL), high low-density lipoprotein cholesterol (LDL-c > 130 mg/dL), and low high-density lipoprotein cholesterol (HDL-c < 60 mg/dL) [24]. The glycemic profile was analyzed using homeostasis model assessment of insulin resistance (HOMA-IR) as a marker of insulin resistance (IR > 2.72) [25] and high fasting insulinemia (25 mU/L) [3], fasting blood glucose, and glycosylated hemoglobin. C-reactive protein (CRP) was classified as nonreactive (<6 mg/L) or reactive (>6 mg/L). The following methods were used in the biochemical analyses—colorimetric enzymatic assay to assess fasting blood glucose and lipid profile, electrochemiluminescence to assess fasting insulinemia and HOMA-IR, immunoturbidimetry to assess glycosylated hemoglobin, and semiquantitative CRP.

The presence of T2DM was defined by the use of hypoglycemic agents and/or the following—fasting blood glucose ≥ 126 mg/dL and glycosylated hemoglobin ≥ 6.5% (ADA, 2019). Systemic arterial hypertension (SAH) was determined when systolic blood pressure was ≥140 mmHg and diastolic blood pressure was ≥90 mmHg and/or by antihypertensive drugs use [26]. Blood pressure (BP) in the brachial artery was measured using the automatic sphygmomanometer Omron HEM-742INT (Omron Healthcare, São Paulo, SP, Brazil), with an appropriate cuff size for severely obese individuals. The mean of two measurements taken at rest 2 to 3 min apart was used in BP classification [27].

### 2.5. Ethical Aspects

This study follows the Declaration of Helsinki for experiments on human beings. All participants were volunteers and provided written informed consent. The study was approved by the Ethics Committee (protocol No. 747,792/2014).

### 2.6. Statistical Analysis

The prevalence and prevalence ratio (PR) were calculated with a 95% confidence interval (CI) for all explanatory variables according to the outcome variable T2DM, using the reference category with the lowest frequency in each variable to calculate the PR [28]. The linear trend chi-square test was used between age and T2DM. The normality of continuous data was calculated using the Kolmogorov–Smirnov test. After confirming the normality of the data, the Student’s *t* test was used to calculate parametric data means; Pearson correlation was used between BMI and the following variables—fasting blood glucose, glycosylated hemoglobin, HOMA-IR, and fasting insulinemia.

Variables with a *p*-value < 0.20 in the bivariate analysis were included in the multivariate analysis using the Poisson regression [28]. There was an adjustment for the use of hypoglycemic agents, with the exception of the variables that are part of the diagnostic criterion of diabetes used to define the outcome of the present study. A *p*-value < 0.05 was considered statistically significant in all analyses.

## 3. Results

This study included 150 class II and III obese individuals, with a T2DM prevalence of 40% (95% CI, 32.1–48.3). The mean age of the study participants was 43.0 ± 4.4 years, and the mean BMI was 48.4 ± 6.1 kg/m^2^. Twenty-seven patients with T2DM (45%) were using oral antidiabetic agents, mainly metformin (*n* = 25). Only three patients were on insulin therapy.

Lower educational levels (PR = 1.93, 95% CI, 1.32–2.80) and age > 50 years (PR = 3.17, 95% CI, 1.26–7.98) were significantly associated with T2DM, with a positive linear trend between age and T2DM (Table 1).

SAH, hypertriglyceridemia, and high CRP level were statistically associated with T2DM in class II and III obesity (Table 2). The prevalence of high fasting glucose was 19.33% (95% CI, 13.3–26.6) and high glycated hemoglobin was 32.67% (95% CI, 25.2–40.8).

The variables included in multiple regression were age and educational level (1st level); consumption of raw vegetables and whole cereals (2nd level); and hypertension, HOMA-IR, fasting insulinemia, hypertriglyceridemia, HDL, and CRP (3rd level). The variables associated with T2DM were lower educational level (*p* = 0.018), nonconsumption of whole grains (*p* = 0.049), and high HOMA-IR (*p* = 0.016) (Table 3).

**Figure 1:**
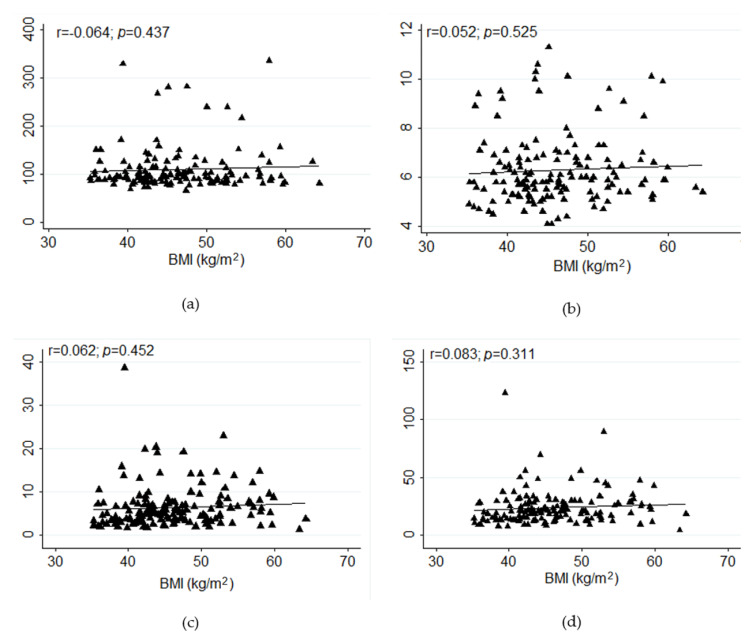
Correlation between body mass index and glycemic parameters in class II and III obesity ((**a**). fasting blood glucose, (**b**). glycosylated hemoglobin, (**c**). homeostasis model assessment of insulin resistance, (**d**). insulin).

There was no significant correlation between BMI and the glycemic parameters fasting blood glucose, glycosylated hemoglobin, HOMA-IR, and insulin (Figure 1). Additionally, in a subgroup analysis, no significant correlation was found between BMI and fasting blood glucose, glycosylated hemoglobin, HOMA-IR, and insulin, respectively—only participants with T2DM (*p* = 0.372, *p* = 0.807, *p* = 0.811, *p* = 0.722), participants with T2DM without antidiabetic agents (*p* = 0.483, *p* = 0.467, *p* = 0.709, *p* = 0.734), and participants with TD2M on antidiabetic agents (*p* = 0.465, *p* = 0.153, *p* = 0.459, *p* = 0.438).

## 4. Discussion

The present study is an important contribution to T2DM and obesity research as it is one of the few studies to evaluate T2DM-associated factors specifically in class II and III obese individuals not having undergone previous bariatric surgery [7]. In addition, this study also fills a gap in the analysis of food consumption and body composition, as well as in the correlation between BMI and glycemic profile, which are variables poorly investigated in the class II and III obese population. The results show a high prevalence of T2DM (40%) in this population, with the associated risks being lower educational level, nonconsumption of whole grains, and high HOMA-IR. Because there are no previous studies on T2DM in class II and III obesity, it is impossible to compare results; however, this emphasizes the relevance of these findings.

The association between lower educational level and T2DM in class II and III obesity corroborates other studies in which the same association was reported [29,30], as well as in individuals with a normal BMI [31]. Lower educational level is an important variable as it can influence the treatment of obesity due to the difficulty in understanding the guidelines or prescriptions proposed by healthcare professionals [29,32,33,34]. Therefore, identifying the appropriate approach and more understandable strategies for those with lower educational attainment as a way to improve clinical treatment and increase compliance is a challenge for the multidisciplinary healthcare team. In addition, lower educational levels are associated with low socioeconomic status, directly implying reduced access to agile and continuous healthcare services and decreased access to healthy food, equipment for regular PA, and participation in regular PA programs [35,36,37,38,39].

This study showed an association between nonconsumption of whole grains daily and T2DM in class II and III obesity. Low whole-grain consumption can be influenced by large supplies of ultraprocessed foods combined with the lower price of these products [40]. It has been reported that whole grains are less accepted, which is related to the limited time and ability to prepare these foods, low financial conditions to purchase them, and low availability of products in the supermarkets [41,42]. Consequently, the reduced consumption of whole grains may also reflect the general quality of the diet, which tends to be low in fiber, playing an essential role in the control of body weight and lipid and glycemic profiles [13,43,44]. Evidence suggests that a diet rich in whole grains and vegetables with reduced consumption of refined grains, sucrose, and fructose may have a protective role against diabetes [13,40,43].

The association between HOMA-IR and T2DM in class II and III obesity identified in this study reinforces the IR status. An increased HOMA-IR is a common feature for T2DM in obese individuals due to a greater production of inflammatory markers by the visceral adipose tissue and lower levels of adiponectin, a condition that favors IR and T2DM [45,46,47]. One cohort study showed that IR obese individuals had more abdominal fat, higher CRP levels, and lower adiponectin levels compared with nonresistant individuals (*p* = 0.015 to <0.0001) [48]. HOMA-IR is one of the most frequently used indicators for T2DM prognosis and control; higher values are associated with a higher risk of diabetic retinopathy, neuropathy, nephropathy, coronary artery disease, and peripheral vascular disease [49,50]. On the other hand, a European study of nondiabetic individuals with different BMI ranges did not find an association between obesity and insulin resistance, where prevalence of insulin resistance was relatively low [51]. This study also show that obesity per se is not the only factor causing diabetes, and it is necessary to have insulin resistance with obesity to develop T2DM [51].

This study showed no association between T2DM and body composition, inflammatory markers, or lipid profile, in contrast to a study by Fronczyk et al. [52], which reported that BMI (*p* < 0.0001) and presence of diabetes (*p* = 0.011) are independent factors increasing CRP levels. As for the lipid profile, compared with nondiabetic obese individuals, obese diabetic individuals presented with increased total cholesterol (*p* < 0.001), triglycerides (*p* < 0.001), and LDL-c (*p* < 0.001) levels [48,53].

Despite the hypothesis that increased BMI correlates with glycemic profile parameters in studies on preobesity [54,55,56], this correlation was not observed in the present study. These results highlight the importance of deepening knowledge about T2DM, specifically in severely obese individuals. A previous study of severely obese individuals reported that increased obesity levels were a factor associated with worsening glucose homeostasis when compared with other obesity classes [7]. Cardiometabolic complications are more prevalent and severe in these individuals than in less obese individuals [7].

One of the limitations of this study is the difficulty in measuring eating habits. However, this is inherent to the food frequency instrument itself, which has been widely used in research in this area [57]. As there is no instrument specifically validated to analyze class II and III obesity, the instrument used in this study was the FFQ, as recommended for population-based studies with adults [58]. In this study, several methodological precautions, such as the training of interviewers and nutritionists, improvement of the questionnaire in a pilot study, and the development of Standardized Operating Procedures for all stages of the research, were taken to ensure the quality of the data collected. The instruments used in the present study, such as triaxial accelerometer and multifrequency BIA, were all previously calibrated and reinforced the quality of the data.

## 5. Conclusions

To summarize, the risk factors associated with a high prevalence of T2DM in class II and III obesity were found to be lower educational level, nonconsumption of whole grains, and high HOMA-IR values. There was no correlation between BMI and glycemic parameters, that is, an increased BMI did not directly affect a decline in the metabolic condition of T2DM individuals. It is important to emphasize the need to perform further research on T2DM in this specific class II and III obese population. Future studies should analyze food consumption, body composition, and metabolic, glycemic, and inflammatory parameters to increase knowledge in this area, contributing to the development of T2DM prevention and the establishment of specific clinical protocols for this population.

## Figures and Tables

**Table 1:** Prevalence of type 2 diabetes mellitus and its associated factors in class II and III obesity with respect to sociodemographic, lifestyle, and food consumption variables.

Variables	Total	Diabetes Mellitus		*p*-Value ^1^
		Prevalence	PR (95% CI)	
	*n* (%)	*n* (%)		
Sex				0.925
Female	128 (85.33)	51 (39.84)	1.00	
Male	22 (14.67)	9 (40.91)	1.02 (0.59–1.77)	
Age ^2^				**0.011**
18–29 years	19 (12.67)	4 (21.05)	1.00	
30–39 years	57 (38.00)	22 (38.60)	1.83 (0.72–4.66)	
40–49 years	53 (35.33)	20 (37.74)	1.79 (0.70–4.59)	
50–64 years	21 (14.00)	14 (66.67)	3.17 (1.26–7.98)	
Color				0.400
White	46 (30.67)	16 (34.78)	1.00	
Pardo/Black	104 (69.33)	44 (42.31)	1.22 (0.77–1.92)	
Marital status				0.313
Without partner	55 (36.67)	19 (34.55)	1.00	
With partner	95 (63.33)	41 (43.16)	1.25 (0.81–1.92)	
Educational level				**<0.001**
Up to 8 years of study	49 (32.67)	29 (59.18)	1.93 (1.32–2.80)	
≥9 years of study	101 (67.33)	31 (30.69)	1.00	
Economic class				0.393
A/B	34 (22.67)	11 (32.35)	1.00	
C	92 (61.33)	37 (40.22)	1.24 (0.72–2.15)	
D/E	24 (16.00)	12 (50.00)	1.54 (0.82–2.91)	
**Lifestyle**				
Smoking habits				0.616
Nonsmoker	101 (67.33)	39 (38.61)	1.00	
Smoker or ex-smoker	49 (32.67)	21 (42.86)	1.11 (0.74–1.67)	
Physical activity level				0.208
<150 min/week	132 (93.62)	49 (37.12)	1.00	
≥150 min/week	9 (6.38)	5 (55.56)	1.49 (0.79–2.80)	
**Food consumption**				
Raw vegetables				0.133
Not daily	81 (54.00)	37 (45.68)	1.37 (0.91–2.07)	
Daily	69 (46.00)	23 (33.33)	1.00	
Cooked vegetables				0.882
Not daily	109 (72.67)	44 (40.37)	1.03 (0.66–1.62)	
Daily	41 (27.33)	16 (39.02)	1.00	
Fresh fruit				1.000
Not daily	110 (73.33)	44 (40.00)	1.00 (0.64–1.56)	
Daily	40 (26.67)	16 (40.00)	1.00	
Whole-grain cereals				0.145
Not daily	126 (84.00)	54 (42.86)	1.71 (0.83–3.54)	
Daily	24 (16.00)	6 (25.00)	1.00	
Sweets and candy				0.815
Not daily	136 (90.67)	54 (39.71)	1.00	
Daily	14 (9.33)	6 (42.86)	1.08 (0.57–2.05)	
Sugary drinks				0.536
Not daily	98 (65.33)	41 (41.84)	1.14 (0.74–1.76)	
Daily	52 (34.67)	19 (36.54)	1.00	
Meal fractionation				0.349
<3 meals/day	31 (20.67)	10 (32.26)	1.00	
≥3 meals/day	119 (79.33)	50 (42.02)	1.30 (0.75–2.27)	

PR, prevalence ratio; CI, confidence interval. ^1^ Chi-square. ^2^ Linear chi-square, *p* = 0.011. **Bold**: *p* < 0.05.

**Table 2:** Prevalence of type 2 diabetes mellitus and its associated factors in class II and III obesity with respect to systemic arterial hypertension, biochemical parameters, obesity class, and body composition.

Variables	Total	Diabetes Mellitus		*p*-Value ^1^
		Prevalence	PR (95% CI)	
	*n* (%)	*n* (%)		
**Arterial hypertension**				**0.002**
No	65 (43.33)	16 (24.62)	1.00	
Yes	85 (56.67)	44 (51.76)	2.10 (1.31–3.78)	
**Biochemical parameters**				
High HOMA-IR				0.109
No	101 (67.33)	36 (35.64)	1.00	
Yes	49 (32.67)	24 (48.98)	1.37 (0.93–2.03)	
High fasting insulinemia				0.093
No	23 (15.33)	5 (21.74)	1.00	
Yes	127 (54.67)	55 (43.31)	1.99 (0.89–4.45)	
Hypercholesterolemia				0.891
No	94 (62.67)	38 (40.43)	0.97 (0.64–1.46)	
Yes	56 (37.33)	22 (39.29)	1.00	
Hypertriglyceridemia				**0.015**
No	81 (54.00)	25 (30.86)	1.00	
Yes	69 (46.00)	35 (50.72)	1.64 (1.10–2.46)	
High LDL-c				0.739
No	113 (76.87)	47 (41.59)	1.09 (0.67–1.76)	
Yes	34 (23.13)	13 (38.24)	1.00	
Low HDL-c				0.118
No	14 (9.33)	8 (57.14)	1.49 (0.90–2.47)	
Yes	136 (90.67)	52 (38.24)	1.00	
CRP				**0.046**
Nonreactive	63 (42.00)	19 (30.16)	1.00	
Reactive	87 (58.00)	41 (47.13)	1.56 (1.01–2.42)	
BMI				0.894
35.0–44.9 kg/m^2^	76 (50.67)	30 (39.47)	1.00	
≥45.0 kg/m^2^	74 (49.33)	30 (40.54)	1.03 (0.69–1.52)	
**Body composition**				
Total body fat, %				0.976
1st and 2nd quartiles	71 (48.97)	28 (49.12)	1.00	
3rd and 4th quartiles	74 (51.03)	29 (50.88)	0.99 (0.66–1.49)	
Total fat mass, kg				0.918
1st and 2nd quartiles	72 (49.66)	28 (49.12)	1.00	
3rd and 4th quartiles	73 (50.34)	29 (50.88)	1.02 (0.68-1.53)	
Fat-free mass, kg				0.293
1st and 2nd quartiles	71 (48.97)	31 (54.39)	1.24 (0.83–1.87)	
3rd and 4th quartiles	74 (51.03)	26 (45.61)	1.00	
	**Total**	**Nondiabetic**	**Diabetic**	***p*** **-value ^2^**
	**Mean ± SD**	**Mean ± SD**	**Mean ± SD**	
Total body fat, %	51.58 ± 4.68	51.32 ± 4.50	51.98 ± 4.97	0.409
Total fat mass, kg	61.44 ± 12.96	61.84 ± 13.42	60.82 ± 12.30	0.647
Fat-free mass, kg	57.32 ± 8.95	58.05 ± 9.52	56.19 ± 7.93	0.221

HOMA-IR, homeostasis model assessment of insulin resistance; LDL-c, low-density lipoprotein cholesterol; HDL-c, high-density lipoprotein cholesterol; CRP, C-reactive protein; BMI, body mass index; PR, prevalence ratio; CI, confidence interval; SD, standard deviation. ^1^ Chi-square. ^2^ Student’s *t*-test. **Bold**: *p* < 0.05.

**Table 3:** Variables associated with type 2 diabetes mellitus in class II and III obese individuals after hierarchical multivariate analysis and adjustment for hypoglycemic agent use.

Variables	Diabetes Mellitus	*p*-Value ^1^
	Adjusted PR (95% CI)	
**1st level**		
Age		
18–29 years	1.00	
30–39 years	1.84 (0.86–3.96)	0.117
40–49 years	1.23 (0.59–2.60)	0.589
50–64 years	1.89 (0.86–4.14)	0.111
Educational level		
Up to 8 years of study	1.49 (1.07–2.09)	**0.018**
≥9 years of study	1.00	
**2nd level**		
Raw vegetable consumption		
Not daily	1.04 (0.73–1.49)	0.822
Daily	1.00	
Whole-grain cereal consumption		
Not daily	1.67 (1.00–2.80)	**0.049**
Daily	1.00	
**3rd level**		
Low HDL-c		
No	1.05 (0.69–1.59)	0.805
Yes	1.00	
High fasting insulinemia		
No	1.00	
Yes	1.29 (0.63–2.65)	0.480
CRP		
Nonreactive	1.00	
Reactive	1.19 (0.79–1.78)	0.401
Hypertension		
No	1.00	
Yes	1.29 (0.82–2.02)	0.270
Hypertriglyceridemia		
No	1.00	
Yes	1.37 (0.96–1.93)	0.077
High HOMA-IR		
No	1.00	
Yes	1.54 (1.08–2.18)	**0.016**

HDL-c, high-density lipoprotein cholesterol; CRP, C-reactive protein; HOMA-IR, homeostasis model assessment of insulin resistance; PR, prevalence ratio; CI, confidence interval. ^1^ Chi-square. **Bold**: *p* < 0.05.
